# The New York Institute of Stomatology

**Published:** 1899-03

**Authors:** 

**Affiliations:** The New York Institute of Stomatology


### THE NEW YORK INSTITUTE OF STOMATOLOGY.

A meeting of the Institute was held Tuesday evening, November 1, 1898, at the office of Dr. George S. Allan, No. 51 West Thirty-seventh Street, the Vice-President, Dr. C. A. Woodward, in the chair.

The minutes of the previous meeting were read and approved.

### COMMUNICATIONS ON THEORY AND PRACTICE.

Dr. George A. Wilson read the following communication from Dr. George L. Parmele, of Hartford.

"Gentlemen,—Over one hundred years have passed away since the first dental operations were performed in America,—probably thousands since ancient races in Central America, drilling the teeth with reeds and sand, inserted inlays of jade; nearly sixty years have departed since at Baltimore the degree of Doctor of Dental Surgery was given to the world, and thirty years since the degree, the abbreviation for which is D.M.D., was conferred by Harvard University, and later by the University of Minnesota. We have now in the United States in the vicinity of twenty thousand dentists, and dental colleges galore; so, surely the time has arrived when it may be proper to call attention to the fact that, as a rule, the abbreviations for these degrees do not appear in the dictionaries, although many others, less commonly employed, do. I have examined the best authorities, which I am told are the following:

"*Century Dictionary* gives D.D.S., but not D.M.D.

"*Webster*, edition 1890, gives 'D.M.D. (Doctor Medicinæ Dentatis), Doctor of Dental Surgery' (?), but it does not give D.D.S.

"*Worcester*, edition 1865, gives neither.

"*Standard*, edition 1896, gives neither.

"I was told that I might find them in 'Hill's Annual,' but search failed to reveal them. Can we as a scientific body do something to place ourselves on record on this point?"

*Dr. S. A. Hopkins.*—I have been informed that the Century Company is now making a supplementary edition of its Dictionary; so that if anything is to be done, now is the time to do it. They are not to change the plates of the original, but they will publish a supplemental edition containing the omissions of the original.

*Dr. J. Morgan Howe.*—I move that a committee be appointed by the chair to take this communication into consideration.

Carried.

*The Chairman.*—It is an unexpected pleasure to announce that we have among us Dr. S. A. Hopkins, of Boston, who has something of interest for us.

*Dr. Hopkins.*—Mr. President and Gentlemen, I have here a few cultures of chromogenic, or color-producing, bacteria that have been taken from the human mouth. So far as I can learn, most of these forms have not been as yet described, and yet they are not very uncommon. This work on chromogenic bacteria is incidental to other lines of study. It does not bear, perhaps, any very clear relation to our clinical work, but it may be interesting to you to hear about these things, as they cause a good deal of the discoloration which takes place in the mouth, and because they are not described in the usual authorities. Of course, my bacteriological work was undertaken with the idea of studying the pathogenic forms, those which produce the various diseases of the mouth, but these chromogenic forms came cropping up in such numbers that it was impossible to neglect them.

I have brought with me only those forms which are yellow, or might be classified under the yellow head, with the exception of the prodigiosus, a form with which you may be familiar. I have isolated from the mouth twelve different forms of chromogenic bacteria which produce the yellow color, and I think at least eight of those forms have never been described.

This one, marked "No. 1 A," is a bacillus, which stains readily by all the aniline stains, and has shown slight pathogenic properties. Inoculated into a guinea-pig it produces a certain amount of pus, but it does not kill the pig; it is probably harmless in healthy mouths.

"No. 1 B" is a short motile rod, which grows both in the incubator and at the room temperature, does not produce spores, and does not liquefy gelatin. This came from the same mouth that the first form came from.

This one is a yellow coccus without pathogenic properties, a common form, which has been described before.

This test-tube contains a form which was rather interesting because it was very much like the staphylococcus pyogenes aureus, which produces large abscesses when inoculated into guinea-pigs, and is probably responsible for many abscesses in the human subject.

This form is not the staphylococcus pyogenes aureus, because it does not coagulate milk and does not stain by Gram's method. It is interesting because it was taken from a woman about to be confined, and I found that it killed a guinea-pig in about forty-eight hours with rather violent symptoms and a good deal of diarrhœa. I rushed around to the woman's physician and told him the facts of my discovery, feeling that he ought to be informed. He exclaimed, "I wish it would have the same effect on her; I have been giving her quarts of laxatives, and I cannot get the slightest movement of the bowels." It does not always follow that a form that is pathogenic for lower animals is necessarily so for human beings. This woman is still in perfect health, though her mouth always contains this form.

Here is another one. This is rather a beautiful mass; this coccus form is seen very often on the teeth at the margin of the gums in mouths that are not well cared for.

Here is another form which approximates the one I have just given you.

This form is interesting from two points of view,—first, because of its variable morphology; and second, because of the fact that it does not grow at the body temperature; it grows very well at the room temperature, and will develop a considerable growth at a temperature of 28° F. That was exceedingly interesting, because it was undoubtedly a mouth form. I found it first in one mouth, three months after in the same mouth, and again, a year later, in another mouth. It is not, perhaps, a common mouth form, but it is interesting because it does not grow at the body temperature and will develop even when the temperature is below freezing, which seems peculiar for a mouth form. It points perhaps to the habit of mouth-breathing. It is also interesting because its morphology is exceedingly variable. It grows from a rod form to a form which would readily be taken for a coccus, and from the coccus form to an unmistakable rod. The rod form is best obtained by cultivating on potato, while the coccus form is acquired when cultivated on old dry blood serum tubes. I have given the name of "Micrococcus Subnormalis" to this bacterium, and if any gentleman wishes to know more about it and will send me his address, I will gladly send him a little brochure on the subject.

Here is another culture, a beautiful rod. I think this is probably the bacillus aureus, although the descriptions in the books are so confusing that it is not quite possible to determine.

These are also forms without pathogenic properties.

This No. 12 has certain pathogenic properties. It produces pus when inoculated into white mice.

Here are some forms which I have recently found and not yet classified.

This is the bacillus prodigiosus, with which I think you are all familiar. It is a large rod, as you know, and at one time was prevalent all over Europe. In this bacillus we find the explanation of the miracle of the bloody bread which astonished the credulous and excited faithful worshippers all over the continent of Europe. The bread was exposed in the cathedrals, and after twenty-four or forty-eight hours had passed, by reason of the growth of this bacillus prodigiosus, it appeared red upon the surface. This color was supposed to be produced by the blood of Christ.

*Dr. George S. Allan.*—With the exception of a monograph by Dr. Miller, of Berlin, published two or three years ago, on the pathogenic bacteria of the mouth, I know of no other article obtainable on the subject, and this only briefly refers to the chromogenic bacteria of the mouth. These cultures are certainly remarkably perfect and show the characteristic appearances, including color of the growing bacteria *en masse*—mixing beautifully.

While I must confess my ignorance of the subject, I rise to thank the doctor for bringing on and showing us the results of his careful work. Of course, we all recognize the fact that while caries of the teeth is undoubtedly of germ origin, none of the so-called chromogenic bacteria have anything to do with the decay of the teeth.

*Dr. Hopkins.*—It is perfectly possible to produce decay artificially, but, so far as I know, nobody has been able to do so with pure cultures.

Dr. W. H. McCutcheon presented a paper entitled "Two Cases of Diseased Antrum."

(For Dr. McCutcheon's paper, see page 163.)

DISCUSSION.

*Dr. Charles O. Kimball.*—I only rise to call attention to the suggestion made at a meeting of the Institute some two or three years ago by Dr. Dawbarn. He suggested that in these antrum cases, instead of removing the tooth we should go high up under the lip and reach the antrum above the root of the tooth, through the external wall of the antrum at its lowest point, where the bone is very thin. He said he had practised that himself, and it had proved very successful, not interfering with the teeth in any way, affording perfect drainage, and being more easily kept clean than when the opening was made in the customary way through the socket of the tooth.

*Dr. E. H. Raymond.*—These cases are exceedingly interesting to me, because I have had some experience in treating antral diseases. Examination of a number of skulls will show that roots of the superior bicuspids and the molar teeth often penetrate the floor of the antrum. If the pulps of these teeth die, you know what to expect. If we bore from the side, it will, it seems to me, be difficult to get perfect drainage, remove the cause of trouble, and get the antral cavity clean, except in special cases.

In 1894 a young lady, living in Connecticut, had been ill for nearly a year, and the physicians failed to discover the cause of her illness. Her friends sent her to the city to consult a specialist. I referred her to Dr. Abbott, but she asked me to do what I could to relieve her. She had lost about thirty pounds in weight. I diagnosed the case as "antral disease." As she had had a second molar extracted some months previous to her coming to New York, I drilled through this space into the floor of the antrum. I used cocaine freely, and gave her scarcely any pain. It was necessary to open the windows when I got through. After syringing freely with warm water, I used listerine. I then gave her a syringe, with instructions to use diluted listerine three times a day through the opening, and to report in a week. She reported having had a good appetite, refreshing sleep, and felt herself to be a new creature. She has never had a recurrence of the trouble, and her health was soon re-established.

I have treated several cases since with the same gratifying results. Removing the cause of the trouble and getting perfect cleanliness is all that is necessary in order to restore normality, in most cases.

*Dr. S. E. Davenport.*—I consider that Dr. Raymond was very fortunate in that his patient had lost a tooth, through the old socket of which he was able to drill and get proper drainage without the responsibility of causing the patient to lose a tooth. I was very much interested in Dr. McCutcheon's reports. It seems to me that he treated the pathological conditions with skill and certainly with success, but I feel like commiserating with him upon the apparent necessity of extracting so many teeth. Dr. Dawbarn, who was kind enough to demonstrate upon the cadaver many little things for us a year or two ago, and to whom Dr. Kimball has referred this evening, rather overturned our ideas of general surgeons practising so-called oral surgery, for our experience in past years with general surgeons in such cases has been that the gentlemen operating did not seem to consider teeth at all; they seemed to look upon the teeth as simply pegs in a hole, which it was well to get rid of for the sake of the hole, which would thereafter be useful; neither did they appear to care about the expression of the face after they had finished, so long as they had cured the trouble. But as I have said, Dr. Dawbarn showed us upon the cadaver the very best point by which to reach the antrum, the thin wall, and the lowest point of the antrum in many cases. It is most interesting that a general surgeon should encourage the retention of teeth by showing dentists a way to reach and properly drain the antrum in these cases, and, perhaps, I may be forgiven for adding that it is deplorable that dentists are so easily persuaded, when pressed by patients, to extract teeth for the treatment of this trouble.

*Dr. Howe.*—I would like to say that from my observation antra vary very greatly in size and shape. I have had a case of empyema of the antrum, associated with a lateral incisor, and I entered the sinus through the socket of that tooth. Our chairman told me several years ago of a case in which he entered through the socket of a third molar. I suppose that the best place to open into the antrum may be different in different cases. A seriously diseased tooth and socket may make the loss of the tooth necessary, and in that case the socket of the tooth may be the best place for us, under the circumstances, to make the opening.

*Dr. R. H. M. Dawbarn.*—I am very sorry that I was not able to come, as I meant to, in time to hear the paper of the evening and the points which were brought out. I would say that the way in which I determine where I shall penetrate the bone is by the use, almost always, of the electric light. It is called a "Mignon" lamp, being not much larger than a pea; it is to be put into the patient's mouth in a dark room. The general effect, as to the appearance of the patient's face while the light is burning, is that of a "pumpkin lantern." On the unaffected side it lights up the face beautifully by a somewhat triangular spot of light, nearly an inch across, below the orbit. This can be seen with ease for many yards; but on the side in which the antrum is filled with a new growth or with a turbid fluid, the face will be dark. Now, it is commonly true, though not always do we find the rule hold good, that the anterior edge of the antrum about corresponds with a point a little behind the canine and goes back to a point a little in front of the last molar. It is most closely in relationship with the first and second molars. Should we have an anomaly, the light will indicate the limits of the antral cavity, wherever they may be. It is a fair assumption that if you find an anomaly as to the position of the antrum on the light side, it is likely to be duplicated on the other, or dark side, and you have a good guide in that way.

It seems to me that there can be no question that the old-fashioned method of ruining one of man's best friends, a tooth, even partially sound, for the purpose of entering the antrum ought to be given up. The method of going up through the socket is archaic; it has several disadvantages in addition to the loss of the tooth. One is that you are going through a considerable amount of bone in the alveolar process in order to enter the cavity, and therefore cannot so thoroughly examine the interior of that cavity as you should, and you cannot easily make so large an opening as may at times prove necessary. I am regularly in the habit of making an opening nearly as large as the end of my little finger, and you cannot do that without the loss of a good deal of valuable bone if you go through the socket.

Again, if you go up through the socket, then in the act of chewing you tend to force food up into the cavity by direct pressure, whereas if you go in above the alveolar process, the lip protects the opening in the act of chewing, and it is easier for the patient to avoid passing food into the cavity.

Again, the bone above the alveolar process, which is the front wall of the antrum, is almost as thin as paper. It is therefore penetrated here with the utmost ease by the first tap of your chisel. It seems to become even thinner in old age.

I use a short drainage-tube of soft rubber in the cavity, the outer edge of which is attached by a suture to the periosteum; and at least once a day, and better oftener, have the antrum washed out, which can readily be done, if need be, by the patient himself, the fluid in this way passing in at the surgical opening and out at the natural one,—the middle meatus of the nose. In the majority of cases peroxide of hydrogen is used,—a neutral solution, diluting, if it proves irritating, with boric acid solution; you will thus very quickly bring such a suppuration to an end. But if the discharge continues for a considerable period, without any obvious tendency to diminish, then you can make up your mind that there is something more serious at fault,—the disease has invaded the bone itself. You may then have to enlarge your opening and gouge or chisel away the diseased bone. A cavity of that sort is gently packed with a long strip of gauze of some kind,—aristol or nosophen gauze, or one of the silver gauzes,—which is changed daily or on alternate days.

I do not know what points you wished me to touch upon, and these upon which I have spoken are simply practical matters of personal experience.

*Dr. Allan.*—Most dentists in treating diseases of the antrum attempt to wash out the cavity with a syringe that has not enough capacity. Several years ago I got up this syringe, and have used it more or less since then, and even this, though it has a capacity of about two ounces, I do not think is large enough in some cases; at least it is none too large. I have several nozzles, and have found it very useful. This smaller nozzle I had made on purpose to get into the antrum through the socket of the wisdom-tooth.

I had one case of this kind which I thought very rare, having been taught to get into the antrum through the first or second molar or the second bicuspid, and it proved to be a troublesome case. There was no disease or trouble of any kind till the wisdom-tooth had been extracted, and then within two weeks the trouble appeared. There was never any large amount of pus, but it was several years before a final cure was effected.

I have a disarticulated skull that is of considerable value, for it shows that the socket of the wisdom-tooth penetrates the antrum. It appears, then, that one can look for antral trouble from any tooth back of and including the lateral incisor.

Dr. Dawbarn spoke about the clinical value of the electric lamp in such cases. I never have used it, but I can see its value and importance. This small electric lamp was brought to me the other day. It is certainly a most powerful lamp for its size; it is hardly bigger than a pea-point, and it has a special value in that it can be put into either of the nostrils and you can look through from the palate. It is worked by five dry cells, which, as you know, are very cheap, and I should imagine a battery of five would last a long time. Dry cells are, of course, the most economical way of obtaining a current for these purposes.

*Dr. G. G. Platt.*—I have to ask for information. The remarks have been very interesting to me. I have in my time met with some very trying cases, where I was positive that the root of the molar pierced the antrum. I am not anxious to sacrifice teeth, but in one case, that of a lady, I opened the tooth and got some discharge, but not sufficient to give relief. I then extracted the tooth, and immediately pus followed, so that I, too, had to open the window. Now, in that case, was it not advisable to take the tooth out?

Another case, that of a man who was treated for catarrh. The tooth was extracted, and in a few days, perhaps a week, by syringing, it healed, the discharge from the nostrils ceased, and he has had no catarrh since. In that case, from the appearance of the root, I am sure that it pierced the antrum.

*The Chairman.*—I notice that Dr. Dawbarn has brought something with him.

*Dr. Dawbarn.*—The bottles that I have brought have neither of them any bearing on the subject at hand. The first one was brought at the special request of Dr. Cook; and if you think this is inopportune and wasting your time, please say so, and he will not do so any more.

This was an appendix vermiformis, perforated by a large white pin which had been swallowed by a nursing baby, and was in the interior of the child for five weeks, and at the end of that time it killed the child by causing appendicitis. I was called in, after septic peritonitis had developed, and was unable to save the baby's life. The pin, though originally white, shows now the characteristic smooth, black tarnish which iron takes on when exposed for a long time to the action of living flesh. The father assured me that he saw the pin in the child's mouth and that the pin was white. There are deposits around it of calcareous material. In all my appendicitis work this is only the second case where there was a true foreign body found. The other was a case of swallowing a small piece of bone. Almost invariably the contents prove to be hard fæces, sometimes of stony hardness and capable of taking a polish. Again and again we see material resembling in shape and color lemon-pits, orange-seeds, date-stones, etc., and in almost every instance it is nothing but fæces, as immersion in boiling water quickly proves. The idea that seeds are other than excessively rare as a cause of appendicitis is an unfortunate one. A member of this society told me some time ago that he had never swallowed grapes because they caused appendicitis. I told him I did not believe there was a case on record where grape-seeds had produced appendicitis.

Here, in this other bottle, is a rather interesting thing. It is a demonstration of an idea as to the best way of keeping needles and other small instruments that annoy us by rusting. I have taken the trouble to interview almost every member of the New York Surgical Society as to how he kept his needles, and I have made a list of ten different methods. Hardly any two keep their needles in the same way. For instance, a number of them, a majority, I think, keep them sewn into a piece of cloth that is greased; some keep them in alboline, which is only objectionable because oily; some keep them in dry powder, talcum for example, the objection to this being that you have to go fishing for your needles, which are hidden in the powder; some try to dry the air in which they are kept. I have tried that without any satisfaction. I have put a little jar of chloride of calcium near the needles in an air-tight case, and in a few days, as we all know, it will melt down to a syrupy liquid, meanwhile absolutely drying the air about it. Professor R. Ogden Doremus once told me that if one had a piano at the sea-side, the dampness would affect it very quickly; and at the sea-side hotel where he stayed he had once used this plan very effectively, placing this chemical within the piano for forty-eight hours, and meanwhile keeping it wrapped in blankets. He said the tone was for a time fully restored. I tried it for needles, and it did not work; the needles, lying near at hand, did not rust, but took on a peculiar tarnish and ceased to be bright. Another method is to keep them in lysol, but this is turbid, and you cannot easily see the needles; like carbolic acid, too, it has a tendency to blunt the edge of the instruments, but upon frequent opening of the bottle this absorbs water from the air. Some keep them in absolute alcohol; a French surgeon, M. Marechal, some time ago, wrote an article recommending a solution of borax and water; on the strength of his reputation I put some needles in the borax solution and ruined them. A surgeon who was formerly in the navy told me that he used to touch his needles with a little brush dipped in compound tincture of benzoin, and said that if sea-going surgeons would use that on all their tools they would have no trouble. I should imagine that might be so, but it is distinctly a bother.

We have known for years that, in sterilizing our instruments, the cheapest, simplest, and best way is to boil them with a little washing soda, say one per cent. Now, it occurred to me that we might be equally fortunate in keeping our needles in cold water if it were saturated with washing soda. I put some needles in such a solution,—ordinary washing soda in water,—and these are the same needles. I took a knife and carefully scraped off the plating of one of them; the other has the ordinary plating, and you can see that they are as silvery and bright as the day I put them in, more than a year ago. I think I can claim that the method is a success. It might be a convenience, also, for dentists, as a means of preserving their smaller implements free from rust.

*Dr. Elliott.*—I am very glad that Dr. Dawbarn has brought this matter up; at the same time, I do not think it is particularly new. For many years the Coast Survey of the United States has used wire for its deep-sea soundings; this wire is always kept in a tank of water which is saturated with carbonate or washing soda. Many years ago I used it in every-day practice. I even tried to use it in keeping the right angle of our engine clean, for it is apparently impossible to stop its rusting. I have kept it in a solution of soda, but there is an objection: the oil which is in the right angle—and has constantly to be present there—immediately saponifies with the soda, and you get a soap which is rather disagreeable to the patient.

*Dr. Dawbarn.*—I am very much interested in this comment by Dr. Elliott, and in publishing this little suggestion I shall of course incorporate his statements, which certainly substantiate still further the value of the plan. I think it is not known among our New York surgeons. I am reminded, however, of Solomon's saying, "There is no new thing under the sun."

*Dr. Raymond.*—It is quite remarkable to see how a foreign substance like this pin could get as far as the appendix before giving trouble to the little patient, owing to the convolutions of the alimentary canal through which it passed. It brings to my mind a case that caused considerable alarm in my own family. A daughter, about thirteen years old, swallowed a peach-pit while trying to laugh and eat at the same time. My physician suggested that she be fed on the inside of wheat bread, so as to have the gluten form a coating around the pit to assist its easy passage through the canal. She was allowed to eat very little except oatmeal with this bread for six days, at the end of which time the pit passed, being black and slimy. In addition to the bread and oatmeal, I gave her a little sweet oil daily, thinking that might assist.

*Dr. Dawbarn.*—In a little child the duodenum is so small that one might very well worry under such circumstances. I think that the treatment was entirely a proper one, except that I do not see any object to be gained by giving the oil, which would tend to soften up the portion of fæces or food around the pit and enable it to be brought roughly or directly in contact with the wall of the intestines. The usual method is to feed the patient upon potatoes, or other starchy food, and to allow him to remain constipated and let the foreign body go through as slowly as may be. In the case of the child to which I have referred domestic medicine was tried, and the family did not ask a doctor's advice until too late. I am told that they gave the baby a laxative, which is in such a case a dangerous thing.

*Dr. S. C. G. Watkins.*—While this subject is up, I might mention a couple of cases which created considerable interest in my vicinity some years ago. One of them was the case of a child which had convulsions, and the doctors were at a loss as to the cause, although at times the child had been in the habit of putting buttons in its mouth, and they were fearful that it had swallowed some of them. After receiving proper treatment the child passed over twenty articles,—shoe-buttons, brass buttons, safety-pins, straight ordinary pins, hairpins, and other things of that nature; yet lived and was well and healthy afterwards. That was in the city of Orange.

The other was the case of a young lady, who swallowed a twenty-dollar gold piece. It caught part way down her throat, but the physicians could not remove it, and so they punched it down, and in about a week it passed all right.

Dr. F. Milton Smith presented a paper on "Root Treatment: A Positive Method."

(For Dr. Smith's paper, see page 152.)

*Dr. Allan.*—Dr. Smith, if I remember rightly, said there were some roots he did not attempt to fill. He neglected to tell us what treatment, if any, he gave such roots and got into and cleaned them. I would like some information on this, to me, important matter.

*Dr. Hopkins.*—I think that is the best essay on the subject that I have ever listened to, and I am glad to be here. I think it is especially good because of its force and thoroughness. I rather think that in the thoroughness of the author lies its great success. I was going to say that possibly a scientific light might be thrown on the subject in this way. Bacteriologists agree that most of the forms which produce pus are aërobic and require oxygen; that a tooth that is thoroughly filled, as we can imagine it filled by the essayist, would pretty thoroughly exclude the pyogenic forms because of the lack of oxygen; it is also well known that the drying of the cavity as thoroughly as he indicated would be a tremendous safeguard even if no antiseptic were used.

*Dr. Platt.*—My point was this, that in a very few days after the doctor had treated the tooth, he kindly sat down and wrote me, giving me his mode of operation, and I have found that where I thoroughly and conscientiously carried out the rules laid down by him I have had perfect success; my faith has been rewarded every time.

*Dr. Davenport.*—The time is so short, I feel that somebody ought to be speaking every moment that is allowed us. I wish to thank Dr. Smith most heartily for his paper. I consider it rather unfortunate that it should come so late in the evening, rather than as a text for an entire evening, and I hope sincerely that the matter will come up for discussion at some future meeting, because I feel sure that all our guests and members are anxious to say a word upon this subject, which is never completed, and upon which probably most of us could throw some new light. We are always pleased with the assertions of men who agree with us, and think them successful and scientific, and I would like to get in under cover with Dr. Smith, not quite to the extent that he has suggested to us to-night, for I have not had the necessary amount of courage to fill quite as promptly as he does, but I have never feared to remove mechanically as much of the *débris* as possible at the first sitting, and my practice has been to free the canals if possible when the tooth was first opened.

I wish to bring to the attention of the Chair this very important question, and to request that it may come up at another meeting, for I am sure there will be insufficient time this evening to discuss it.

*Dr. Watkins.*—I am glad of the opportunity to hear this paper, and I agree with Dr. Smith in many things he has said. There are a few points, however, in which I do not agree with him, and one particular thing is filling the roots at the first sitting; that I do not believe in. I do believe in removing every part of the *débris* that can be removed at the first sitting, but I would not attempt to fill the cavity at that time. In my opinion it would be much better to treat the tooth once or twice, allowing the medicament to remain for a week or ten days, and then fill the tooth, and in that way avoid the swelling which the doctor has told us about, which sometimes lasts for several days.

Then, again, the doctor said he would not enlarge the pulp-canals. I believe it is good practice to enlarge every canal with a Gates-Gliddon drill if the canal be not large enough for perfect access to treat or to fill; enlarge it as far as it can be enlarged towards the apical foramen. I do not believe in being satisfied with that enlargement which can be obtainable simply with a broach. I have not been successful in carrying the material in that way to the end of the root. I have never been satisfied that it could be done as well that way as if a drill had been passed along the canal.

Another thing: the doctor does not recommend the use of antiseptics until the canals are pretty well, or entirely, cleansed. I believe in using antiseptics from the very beginning, removing all the *débris* from the cavity by syringing with antiseptics and having the broaches and other instruments which enter the canals dipped in antiseptics, and thus prevent the possibility of carrying bacteria into the canals. I agree with him most thoroughly in the use of carbolic acid. As I said years ago, if I could have only one medicine in my office, that medicine would be carbolic acid.

I do not see his object exactly in using bibulous paper for pressing gutta-percha into the canals. I would much prefer natural cotton, treated antiseptically.

The use of gold wire was mentioned also, which many years ago was a favorite method of filling canals by Dr. William Morrison, of St. Louis. His method of using was to taper a piece of gold wire to approximately fit the canal, make a nick about one-quarter of an inch from the end, and after pressing it up in the canal give it a twist and break it, leaving the one-quarter inch of wire in the canal. Lead has been used in the same way.

In working antiseptics into the canals I use a method which I first knew to be used a good many years ago by our friend Dr. James G. Palmer; it is by using a jewellers four-sided broach, and twisting a fibre of cotton on it to use as a piston, to pump the antiseptics to the end of the root, forcing them through the apical foramen.

*The Chairman.*—May we hear from Dr. Starr?

*Dr. A. R. Starr.*—I agree with Dr. Smith in regard to the advisability of filling pulp-canals immediately in certain cases, but not in all. I sometimes give my patients the choice of having the canals filled immediately or of waiting; telling them what the result is likely to be, and that they may have trouble for a day or two if the canals are filled at once. I believe in the thorough use of antiseptics before and during the operation of cleansing and filling the canals. I think, as stated by Dr. Smith, the application of the rubber dam is very essential.

I would like to say just a word in regard to antrum cases. I should have gotten up before, but I did not know that I had the privilege. I wish to say that the first case I had of diseased antrum came from a third molar. In the last case which presented itself to me for treatment nature had made an opening in just the location that Dr. Dawbarn has advised us to make it. This patient came to me with a swollen face, and presented the ordinary symptoms of alveolar abscess. Upon opening into the swelling I found, instead of pus, a sort of thin glairy mucus containing flocculi and some indication of pus. The appearance of the discharge led me to suspect necrosis, or some trouble with the antrum. Upon using the probe, I found the antrum open and could distinctly feel the roughened extremity of a buccal root of the second molar protruding into the antrum. I enlarged the opening in the antral wall, amputated the diseased end of the second molar root, and then washed the cavity well with antiseptics. I saw the patient two or three times after the operation, and noted that the discharge kept diminishing in quantity, was not offensive, and the patient was free from pain. I have not seen him for about three months, but have heard that his mouth is perfectly comfortable.

*Dr. Dawbarn.*—I would like to say that I, as a general surgeon with oral tendencies, have been very much interested in the paper, and that the essayist did himself an injustice when he accused himself of empiricism. Carbolic acid and chloroform are both very excellent means of relieving pain, as is well known.

Again, as to the use of the gold wire, I was a little surprised that the suggestion was not made to use silver wire instead, because of the fact that for the last two years silver has come very prominently to the fore on account of its antiseptic powers. We owe to Professor Crede, the German surgeon, this suggestion, but among the first to use it in this country was Professor Halsted, of the Johns Hopkins Hospital, who treats his wounds with silver-foil, and sometimes dusts them with metallic silver, powdered. It has been proved by experiments upon microbes that metallic silver in contact with microbes is attacked by certain of the toxines, probably of acid reaction, produced by them in their life processes and death decomposition, and is dissolved and makes a very poisonous compound; poisonous, that is, to the microbes, but not to the patient. It is thus possible that metallic silver might be preferable to gold. I do not know of gold having the same power.

*Dr. Howe.*—I approve very heartily of the suggestion that this subject should be made a subject for discussion at some future meeting when there is more time. Immediate disinfection and filling of roots was first suggested, so far as I know, by my distinguished friend Dr. Stockwell, of Springfield. I discussed the subject with him before the State society many years ago. I hope that it may be brought up for discussion at some time during the coming year.

*Dr. Smith.*—I will attempt to reply to my friend Dr. Allan first. As I understood his question, it was, "How do you proceed to clean out these fine roots that you cannot fill?" I distinctly said, I think, in my paper that I did not fill them. I will as distinctly say now that I do not mechanically clean them. I know of no way to do it if they are as fine as that gold wire that I have to fill them with, and my experience has been that roots that are so fine that I cannot get into them with my finest broach do not often cause trouble. In cases when they do, and I cannot get to the end of them, I know of no way to relieve them except by going through from the outside and burring away the end of the root. Occasionally I have done this.

As to the strength of the carbolic acid, I use it as strong as it comes from the druggist, and protect the tongue and gums with vaseline and with pieces of bibulous paper.

Dr. Davenport suggested that it was unfortunate that the paper was read so late; it strikes me that the essayist was fortunate in reading it so late in the evening, since it left so little time for his annihilation.

Dr. Watkins has made one or two suggestions to which I should like to give a passing word; one is as to the originator of wire in root filling; I claim no originality for that. I do not know anything about who originated it. I did give Dr. Perry the credit of having suggested the use of gold wire; a hundred other men may have done the same thing. I do not know.

Dr. Watkins also suggested that lead had been used. I have so read, but I fail to see how it would be possible to draw out a piece of lead to the thinness of three-one-thousandths of an inch and have it of any service to go anywhere near the ends of these fine roots. It would not be of any use to me for the use to which I put the gold wire.

In reference to Dr. Dawbarn's suggestion as to silver wire, it seems to be a very apt one, and I am glad to hear it; if there is any virtue in silver wire I want to know it.

I thank you, gentlemen, for the way in which you have "tempered the wind to the shorn lamb" to-night, and I am glad to find that there are so many who come so nearly to my ideas in practice.

A vote of thanks was unanimously extended to Dr. Hopkins, of Boston.

Adjourned.

S. E. Davenport, M.D.S., D.D.S.,
*Editor The New York Institute of Stomatology.*

